# RACO‐1 modulates Hippo signalling in oesophageal squamous cell carcinoma

**DOI:** 10.1111/jcmm.15811

**Published:** 2020-09-07

**Authors:** Dan Pang, Weilong Wang, Xiaofeng Zhou, Kui Lu, Jinghang Zhang, Zhiguo Chen, Lingchao Wang, Fangfang Shen, Zhen Chen, Sujie Wang, Jinghan Hou, Aijia Zhang, Benjie Lv, Can Gao, Ziyi Yan, Yuhan Hu, Tingmin Chang, Lidong Wang, Xiumin Li

**Affiliations:** ^1^ Department of Gastroenterology The Third Affiliated Hospital of Xinxiang Medical University Xinxiang China; ^2^ Xinxiang Key Laboratory for Molecular Therapy of Cancer Xinxiang Medical University Xinxiang China; ^3^ Department of Pathology The First Affiliated Hospital of Xinxiang Medical University Xinxiang China; ^4^ Department of Human Anatomy Department of Basic Medical Sciences Xinxiang Medical University Xinxiang China; ^5^ Department of Pediatrics the First Affiliated Hospital of Xinxiang Medical University Xinxiang China; ^6^ Xinxiang Key Lab of Translational Cancer Research The Third Affiliated Hospital of Xinxiang Medical University Xinxiang China; ^7^ Department of Pathology School of Basic Medical Sciences Xinxiang Medical University Xinxiang China; ^8^ Department of Gastroenterology The First Affiliated Hospital of Xinxiang Medical University Xinxiang China; ^9^ State Key Laboratory of Esophageal Cancer Prevention & Treatment and Henan Key Laboratory for Esophageal Cancer Research The First Affiliated Hospital of Zhengzhou University Zhengzhou China

**Keywords:** Hippo, Oesophageal squamous cell carcinoma, RACO‐1, Ubiquitin, YAP

## Abstract

Oesophageal cancer is one of the most lethal malignancies worldwide, whereas the 5‐year survival is less than 20%. Although the detailed carcinogenic mechanisms are not totally clear, recent genomic sequencing data showed dysregulation of Hippo signalling could be a critical factor for oesophageal squamous cell carcinoma (ESCC) progression. Therefore, understanding of the molecular mechanisms that control Hippo signalling activity is of great importance to improve ESCC diagnostics and therapeutics. Our current study revealed RACO‐1 as an inhibitory protein for YAP/TEAD axis. Depletion of RACO‐1 increases the protein level of YAP and expression of YAP/TEAD target gene. Besides, RACO‐1 silencing could promote ESCC cell invasion and migration, which effect could be rescued by YAP depletion in ESCC cells. Immunoprecipitation showed that RACO‐1 associated with YAP and promote ubiquitination and degradation of YAP at k48 poly‐ubiquitination site. Our research discovered a new regulator of Hippo signalling via modulating YAP stability. RACO‐1 could be a promising factor, which serves cancer diagnostics and therapeutics in ESCC patients.

## 1 INTRODUCTION

Oesophageal cancer ranks NO. 8 in cancer incidence and cancer‐related death in the world.^1^ Approximately 60% of newly diagnosed oesophageal cancer cases are found in China.^2^ However, oesophageal squamous cell carcinoma (ESCC) is the major subtype of oesophageal cancer in China, whereas adenocarcinoma is the dominant type in Western countries. Although there are more than 300 000 new diagnosed cases in China, the rate of oesophageal cancer varies widely in China mainland. For example, there is significantly higher oesophageal cancer incidence in northern part of Henan province, compared with neighbourhood area.^3^ Besides, the known risk factors such as tobacco, alcohol and lack of nutrition recently progress of genomic‐based sequencing showed that the genetic alternations also play critical roles in the oncogenic process. The genomic sequencing and profiling data showed that the deficit of inhibitory control in Hippo signalling is familiar in ESCC patients.^4^ The cancer biology studies illustrated that silencing or inhibiting YAP/TEAD axis dramatically inhibited ESCC cell growth and invasion.^5^, ^6^ However, the detail of the mechanism that controls Hippo signalling activity, especially YAP/TEAD trans‐activation turnover, is still not totally clear. As the particular role of Hippo signalling in ESCC carcinogenesis, it is important to reveal the adjustment mechanism of YAP/TEAD in ESCC.

Hippo signalling was first detected in Drosophila through genetic screening.^7^ More investigation showed that Hippo signalling functions to the tissue haemostasis and organ size control, which depended on a fine balance between cell proliferation and apoptosis.^8^ Kinase cascade is the key to Hippo signalling. MST1/2 (Mammalian sterile 20–like kinase) and SAV1 functions as a complex to phosphorylate LATS1/2 (large tumour suppressor kinase), which subsequently phosphorylates YAP/TAZ and promotes YAP/TAZ cytosol retention and proteolysis.^9^ In addition, when the Hippo signal is turned off, the unphosphorylated YAP/TAZ shifts to the nucleus, activating some transcription factors such as TEADs.^10^ The over‐activation of YAP/TEAD axis was observed in several human cancers.^11^ The molecular biology studies showed that YAP/TEAD activation contributed to several cancer biological behaviours, such as tumour growth, cancer invasion, and ‘stemness’ maintenance.^12^ In ESCC studies, Hippo signalling abnormalities, such as YAP gene amplification and FAT mutations were observed in 40% of oesophageal tumour cases.^4^ YAP level is increased in oesophageal cancer samples and correlates with cancer metastasis and advanced cancer stage.^13^

As the critical role of Hippo signalling in oesophageal cancer, further understanding of the control of Hippo signalling and YAP turnover are of great importance to improve ESCC cancer diagnosis and therapy. Here, we identified a novel inhibitor RACO‐1 for YAP/TEAD axis of Hippo signalling in ESCC. RACO‐1 (RING domain AP‐1 co‐activator‐1, RING finger 187) is a RING finger protein, which functions as an E3 ubiquitin ligase in several cellular processes.^14^ Our current study identifies RACO‐1 as an endogenous inhibitor for YAP/TEAD axis of Hippo signalling in ESCC. RACO‐1 promotes YAP poly‐ubiquitination and degradation, which subsequently leads to transcriptional suppression of YAP/TEAD target genes in ESCC.

## 2 MATERIAL AND METHODS

### 2.1 Cell culture

NEC, EC9706 and HEK293 cells were derived from American Type Culture Collection (ATCC). HEK293 and NEC cells were maintained in Dulbecco's modified Eagle's medium (DMEM, 01‐052‐1ACS, Biological Industries) supplemented with 10% foetal bovine serum (FBS, 10091148, Gibco). EC9706 cells were cultured in Roswell Park Memorial Institute‐1640 (RPMI, 01‐100‐1ACS, Biological Industries) supplemented with 10% foetal bovine serum (FBS, 10091148, Gibco). All cells were cultured in a humidified incubator at 37°C with 5% CO2. All cell lines used in this study were identified.

### 2.2 Plasmids and siRNA

The Myc‐RACO‐1 plasmid was acquired from Origene. The HA‐K48 and HA‐K63 Ubi plasmids were acquired from Henan Collaborative Innovation Center of Molecular Diagnosis and Laboratory Medicine.^15^ The Lipofectamine 2000 (11668019, Invitrogen) was used to transfect plasmid. Small interfering RNAs were used to knock down specific genes. The sequences of RACO‐1 siRNA were shown here: GUGAUGGACCGUAGGAAGAdTdT; UCUUCCUACGGUCCAUCACdTdT and CACUGAGCGGUUCAGGUCAdTdT; UGACCUGAACCGCUCAGUGdTdT. The YAP siRNA sequences were as follows: GCUCAUUCCUCUCCAGCUUTT; AAGCUGGAGAGGAAUGAGCTT. The negative control siRNA sequences were as follows: UUCUCCGAACGUGUCACGUTT; ACGUGACACGUUCGGAGAATT. The RNAiMAX reagent (13778150, invitrogen) was used for siRNA transfection.

### 2.3 RNA extraction and qPCR analysis

RNeasy Plus Mini Kits were used to extract total RNA (Qiagen). Real‐time PCR was performed as previously described.^16^ 36B4 was used for internal control.^17^ The primer sequences were as follows: RACO‐1: F: agg act tga atg acg ccc g; R: tcc atc acg tgt ccc ttc ca. 36B4: F: ggc gac ctg gaa gtc caa ct; R: cca tca gca cca cag cct tc. CTGF: F: ctc gcg gct tac cga ctg; R: ggc tct gct tct cta gcc tg. CYR61: F: agc agc ctg aaa aag ggc aa; R: agc ctg tag aag gga aac gc.

### 2.4 Quantification of cell viability

EC9706 cells were transfected with siRACO‐1 or siControl in 12‐well plates. After transfection for 24 hours, cells were collected and counted. Five thousand cells per well were placed into 96‐well plates. Cell proliferation was measured using the WST‐1 as described previously. ^18^ Absorbance at 450 nm (OD450) was measured at the same time‐point.

### 2.5 Wound‐healing assay

RACO‐1 siRNA or siControl was transfected in EC9706 and NEC cells. After 24 hours, the cells were placed in a 12‐well plate using a culture medium containing 1% FBS. When the cells grow fully, use the tip of a pipette scratch directly. Take photos at the specified time to record the healing and measure the distance of the wound. Wound healing is calculated as follows: [1‐(Width of the wound at a given time/width of the wound at t = 0)] × 100%.

### 2.6 Transwell assay

Cell migration test was performed by Transwell chamber as before. EC9706 cells and NEC cells were both transfected with siRACO‐1 or siControl, and were used in the migration experiment. Stimulating cell migration through starvation, medium containing 10% foetal bovine serum was added to the bottom of the chamber and serum‐free medium was added to the chamber. Twelve hours later, migrating cells were immobilized, stained with 1% crystal violet and counted in three random fields with an optical microscope.

### 2.7 Western blotting

Cellular proteins were extracted by RIPA buffer. Proteins were isolated by 10% sodium dodecyl sulphate‐polyacrylamide gel electrophoresis (SDS‐PAGE) and transferred to a polyvinylidene fluoride (PVDF) membrane. The membrane was sealed at room temperature for 2 hours and then incubated with a primary antibody at room temperature for 2 hours or 4° overnight.

The antibodies used in this study were listed here: Anti‐RACO‐1 (HAP030098, Sigma); Anti‐YAP (SC‐101199, Santa Cruz); Anti‐HA (AB0004, Abways); Anti‐myc (ab9106, Abcam); and Anti‐Actin (GB12001, Servicebio). Membranes were washed and then incubated in secondary antibodies peroxidase‐conjugated AffiniPure Goat Anti‐Mouse IgG or Goat Anti‐Rabbit IgG. Enhanced chemiluminescence system was used to observe the cell membrane.

### 2.8 Co‐immunoprecipitation assay

IP lysate (containing protease inhibitors) was used to extract cell protein. The EC9706 cells proteins were immunoprecipitated with Protein A/G Agarose beads (P2006, Beyotime) and Anti‐YAP (SC‐101199, Santa Cruz)/Anti‐RACO‐1 (HAP030098, Sigma) or Anti‐IgG antibodies overnight at 4°C and detected by the Western blot.

### 2.9 Protein stability assays

HEK293 cells were cultured in 24‐well plates and transfected with 0.5 μg Myc‐RACO‐1 or Myc vector. After 36 hours, cells were treated with 20 μmol/L MG132 (474790, LABLEAD) reagent, and the stability of YAP was detected by Western blot assay. Oesophageal cancer cells were also cultured in 24‐well plates and transfected with siRACO‐1 or siControl. After 36 hours, cells were treated with 100 μmol/L CHX (C7698, Sigma) reagent, and the stability of YAP was detected by Western blot assay.

### 2.10 Poly‐ubiquitination detection assay

To directly detect the enriched K48‐ubiquitinated and K63‐ubiquitinated YAP from the cell extracts, HEK293 cells were transfected with 0.8 μg K48 Ubi or 0.8 μg K63 Ubi plasmids together with 0.8 μg Flag‐YAP plasmid and 0.8 μg Myc‐RACO‐1 or Myc vector. After 24 hours, the cells were treated with 20 μmol/L MG132 for 7 hours, and then, the total protein was extracted and pre‐cleared with 30 μL protein A (Santa Cruz, SC‐2001) for 4 hours. Supernatant was collected and immunoprecipitated with YAP antibody. Western blot with HA antibody was used to detect K48 or K63 poly‐ubiquitinated YAP.

### 2.11 Immunofluorescence assay

EC9706 cells were fixed with 4% paraformaldehyde for 10 minutes, 0.5% Triton perforated for 5 minutes, after that rinsed with PBS for three times and then sealed with 5% BSA at room temperature for 1.5 hours. Rabbit anti‐RACO‐1 polyclonal antibody (HAP030098, Sigma) and mouse anti‐YAP monoclonal antibodies (SC‐101199, Santa Cruz) were used to incubated and then combined with fluorescence secondary antibody (Invitrogen). As negative controls, the samples were incubated with the secondary antibodies without primary antibodies. After stained with DAPI, images were observed under laser scanning confocal microscopy (Nikon C2+/si+ Japan).

### 2.12 Statistics

Student's *t* test and Pearson's correlation coefficient were used for comparisons. A *P*‐value of < .05 was considered to be significant.

## 3 RESULTS

### 3.1 RACO‐1 depletion promoted invasion and migration in ESCC cells

**Figure 1:**
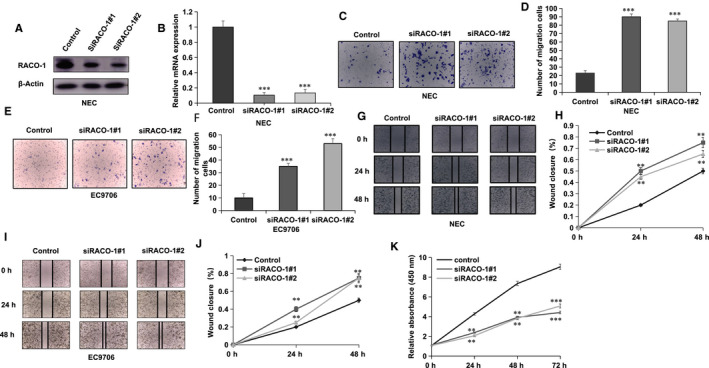
The migration and invasion capacities were inhibited by RACO‐1 in oesophageal squamous cell carcinoma. A and B, The knocking‐down efficiency of RACO‐1 in ESCC cell lines. NEC cells were transfected with RACO‐1 siRNA. After 48 h, RACO‐1 protein and mRNA levels are checked by Western blot and qPCR analysis. Actin was used as internal control. C and D, RACO‐1 depletion increased ESCC cell migration capacity in NEC cells. Two independent siRNA were used in the experiment. Transwell was used to check the migration capacity. The cell number was counted, and data are presented as ±SD. **P < .01, ***P < .001 (Student's *t* test). E and F, RACO‐1 depletion increased ESCC cell migration capacity in EC9706 cells. Two independent siRNA were used in this study. Transwell was used to check the migration capacity. The cell number was counted, and data are presented as ±SD. **P < .01, ***P < .001 (Student's *t* test). G and H, Wound‐healing assay of NEC cells were transfected with indicated 50nM RACO‐1 siRNA (mix of #1 and #2) or 50 nmol/L control siRNA. Quantification of wound closure at the indicated time‐points. Data are presented as ±SD. **P < .01, ***P < .001 (Student's *t* test). I and J, Wound‐healing assay of EC9706 cells were transfected with indicated 50 nmol/L RACO‐1 siRNA (mix of #1 and #2) or 50 nmol/L control siRNA. Quantification of wound closure at the indicated time‐points. Data are presented as ±SD. **P < .01, ***P < .001 (Student's *t* test). K, RACO‐1 depletion inhibits proliferation of ESCC cells. EC9706 was transfected with siControl or siRACO‐1. After 24 h, the WST assay was used to determine the cellar metabolic activity at indicated time‐points after infection. Experiments were done in triplicates. *P < .05, **P < .01, ***P < .001 for cell growth comparison

The NEC and EC9706 cells were used to carry out most of the experiments to illustrate the role of RACO‐1 in ESCC cells. RACO‐1 was depleted in NEC cells via two independent siRNAs to avoid off‐target effect. The knocking‐down efficiency was shown in Figure 1A and B. In order to measure cancer cell migration capacity, transwell assay was carried out in EC9706 and NEC cells. The transwell assay showed that RACO‐1 depletion significantly increased migration capacity in EC9706 and NEC cells (Figure 1C‐F). The wound‐healing assay illustrated that RACO‐1 depletion accelerated the wound‐healing speed in both EC9706 and NEC cells (Figure 1G‐J). However, RACO‐1 depletion inhibited ESCC cell proliferation, which is consistent with previous studies.^14^

### 3.2 RACO‐1 inhibited YAP/TEAD axis of Hippo signalling in ESCC cells

**Figure 2:**
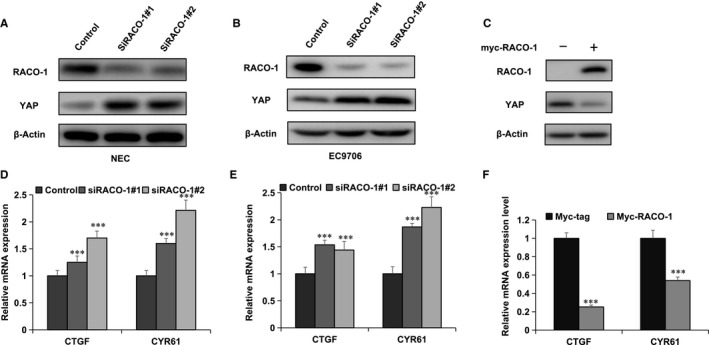
Hippo signalling was inhibited by RACO‐1 in oesophageal squamous cells. A, YAP protein level was increased by RACO‐1 depletion in NEC cells. NEC cells were transfected with siControl or siRACO‐1. After 48 h, cells were harvested for Western blot analysis. RACO‐1 and YAP levels were determined by Western blot. Actin was used as internal control. B, YAP level was increased by RACO‐1 depletion in EC9706 cells. EC9706 cells were transfected with siControl or siRACO‐1. After 48 h, cells were harvested for Western blot analysis. RACO‐1 and YAP levels were determined by Western blot. Actin was used as internal control. C, RACO‐1 overexpression decreased YAP level in EC9706 cells. EC9706 cells were transfected with Myc‐RACO‐1 or Myc plasmids. After 48 h, cells were harvested for Western blot analysis. Myc‐RACO‐1 and YAP levels were determined by Western blot. Actin was used as internal control. D, RACO‐1 depletion increased YAP target gene expression in ESCC cells. NEC cells were transfected with siControl or siRACO‐1. After 48 h, total RNA was extracted for gene expression analysis. Each group was done in triplicates. *P < .05, **P < .01, ***P < .001 for target gene expression comparison. E, RACO‐1 depletion increased YAP target gene expression in ESCC cells. EC9706 cells were transfected with siControl or siRACO‐1. After 48 h, total RNA was extracted for gene expression analysis. Each group was done in triplicates. *P < .05, **P < .01, ***P < .001 for target gene expression comparison. F, RACO‐1 overexpression decreased YAP target gene expression in EC 9706 cells. EC9706 cells were transfected with Myc‐RACO‐1 or Myc plasmids. After 48 h, total RNA was extracted for gene expression analysis. Each group was done in triplicates. *P < .05, ** *P* < .01, ***P < .001 for target gene expression comparison

Then, we analysed the role of RACO‐1in Hippo signalling activity in ESCC cells. RACO‐1 depletion could increase the protein level of YAP in EC9706 and NEC cells (Figure 2A,B). While transient overexpression ofRACO‐1 reduced the protein level of YAP in EC9706 cells (Figure 2C). We further checked the classic target gene of YAP in ESCC cells. Our qPCR results indicated that RACO‐1 depletion could enhance YAP target gene expression (CTGF and CYR61) in both EC9706 and NEC cells (Figure 2D and E). Accordantly, RACO‐1 overexpression decreased the expression of YAP target genes in EC9706 cells (Figure 2F).

### 3.3 RACO‐1 inhibited ESCC cell migration and invasion through Hippo/YAP signalling

**Figure 3:**
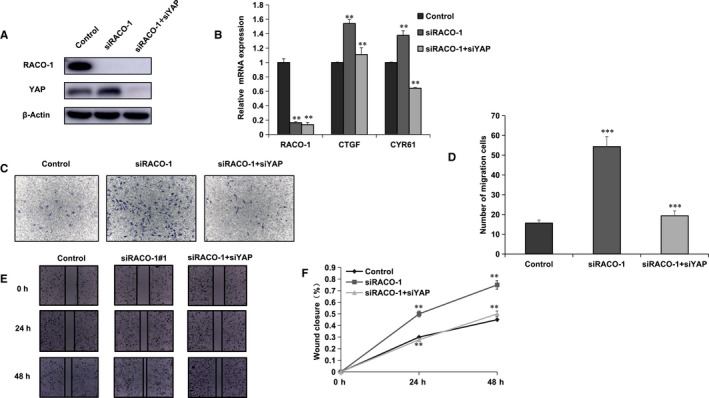
RACO‐1 inhibits cell migration and invasion through Hippo/YAP signalling in ESCC cells. A, The increased YAP protein level by RACO‐1 knocking down could be rescued by YAP depletion. EC9706 cells were transfected with siControl or siRACO‐1. After 24 h, cells were transfected with siYAP or siControl. After 48 h, cells were harvested for Western blot analysis. RACO‐1 and YAP levels were determined by Western blot. Actin was used as internal control. B, RACO‐1 depletion increased Hippo target gene expression, which effect could be reversed by YAP knocking down. EC9706 cells were transfected with siControl or siRACO‐1. After 24 h, cells were transfected with siYAP or siControl. After 48 h, total RNA was extracted for gene expression analysis. Each group was done in triplicates. *P < .05, **P < .01, ***P < .001 for target gene expression comparison. C and D, RACO‐1 depletion increased ESCC cell migration capacity, which effect could be reversed by YAP knocking down. EC9706 cells were transfected with siControl or siRACO‐1. After 24 h, cells were transfected with siYAP or siControl. After another 24 h, cancer cells were seeded into the chamber for transwell assay. The cell number was counted and Data are presented as ±SD. **P < .01, ***P < .001 (Student's *t* test). E and F, Wound‐healing assay indicated that RACO‐1 depletion increased ESCC cell migration capacity, which effect could be reversed by YAP knocking down. EC9706 cells were transfected with siControl or siRACO‐1. After 24 h, cells were transfected with siYAP or siControl. Quantification of wound closure at the indicated time‐points. Data are presented as ±SD. **P < .01, ***P < .001 (Student's *t* test)

Previous data showed that RACO‐1 inhibited YAP level and its target gene expression together with Hippo signalling‐related phenotype. We further investigated whether the change in invasion and migration of ESCC by RACO‐1 knocking down is due to the change of Hippo signalling. Figure 3A indicated that the increased YAP level by RACO‐1 knocking down could be rescued while YAP was silenced. Besides, RACO‐1 depletion could also increase Hippo target gene expression, which could be rescued by YAP depletion in ESCC cells (Figure 3B). We further carried out the experiments of transwell and wound‐healing assays. Consistently, the increased cell invasion and migration by RACO‐1 consumption in ESCC cells could be rescued by further YAP silencing (Figure 3C‐F).

### 3.4 RACO‐1 inhibited YAP stability in ESCC cells

**Figure 4:**
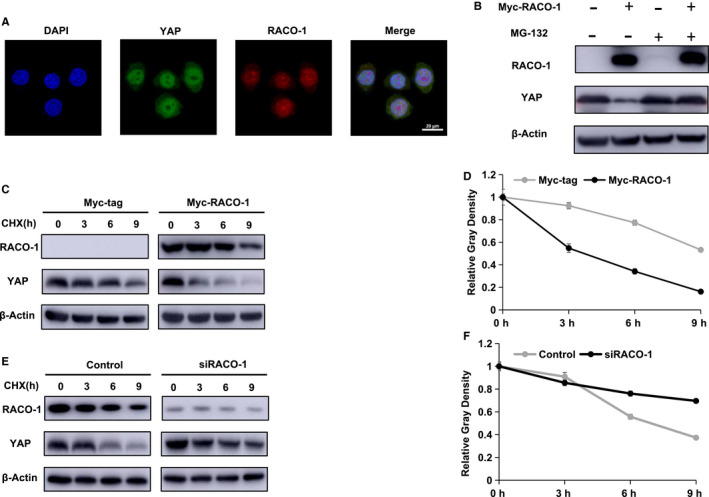
RACO‐1 promotes YAP degradation. A, The localization of RACO‐1 and YAP was analysed in ESCC cells by immunofluorescence assay. EC9706 cells were cultured in normal medium before fixation. Intracellular localization of YAP (green) and RACO‐1 (red) were shown. Nuclei (blue) were stained with 4′,6‐diamidino‐2‐phenylindole (DAPI). B, The degradation effect of RACO‐1 on YAP did not further increase YAP level in the presence of the proteasome inhibitor MG132. HEK293 cells were transfected with 0.5 µg Myc‐tag or Myc‐RACO‐1 plasmids. After 24 h, cells were treated with 20 µmol/L MG132/vehicle for 7 h. Cell lysates were prepared for Western blot analysis. The results are representative for three independent experiments. C and D, YAP half‐life was decreased by RACO‐1 overexpression in HEK293 cells. HEK293 cells were transfected with 0.5 µg Myc‐RACO‐1 or Myc plasmids. After 24 h, cells were treated with 100 µmol/L cycloheximide/vehicle for indicated times. Cell lysates were prepared for Western blot analysis. The results are representative for three independent experiments. The YAP relative density was measured by ImageJ software. E and F, RACO‐1 depletion increased YAP half‐life in EC9706 cells. EC9706 cells were transfected with 50 nmol/L siControl or siRACO‐1. After 24 h, cells were treated with 100 µmol/L cycloheximide/vehicle for indicated times. Cell lysates were prepared for Western blot analysis. The results are representative for three independent experiments. The YAP relative density was measured by ImageJ software

The position of RACO‐1 and YAP were determined in ESCC cells via immuno‐staining, which indicated that both of the proteins located in the nuclear (Figure 4A). RACO‐1 overexpression could decrease the protein level of YAP, whereas the proteasome inhibitor MG132 reversed its role in HEK293 cells (Figure 4B). This phenomenon might indicate that RACO‐1 affect YAP level via post‐translational mechanism. We further measured the protein stability via cycloheximide, a protein synthesis inhibitor. RACO‐1 overexpression in HEK293 cells significantly decreased YAP half‐life (Figure 4C,D). Besides, RACO‐1 depletion could dramatically increase endogenous YAP stability in EC9706 cells (Figure 4E,F).

### 3.5 RACO‐1 interacts with YAP and promoted YAP poly‐ubiquitination

**Figure 5:**
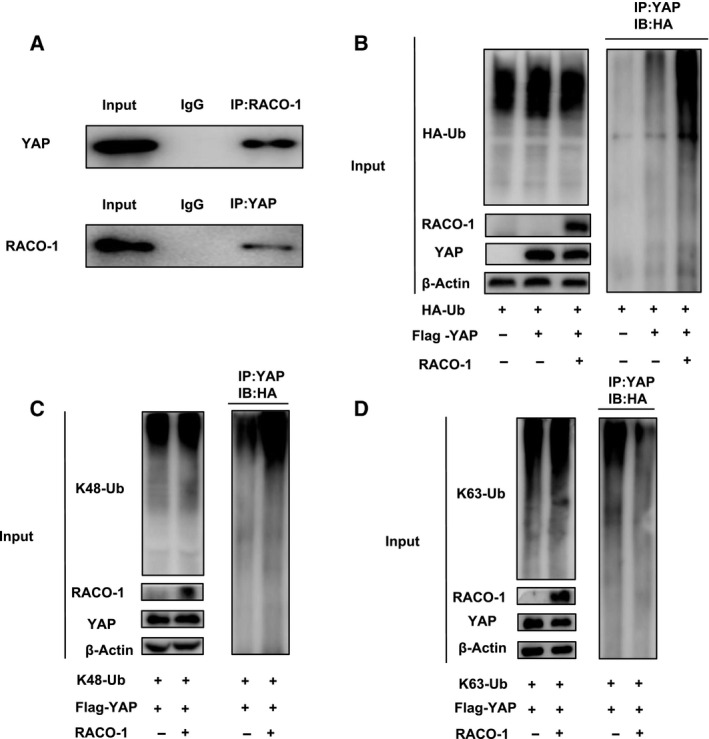
RACO‐1 associates with YAP and promotes YAP K48‐linked poly‐ubiquitination. A, CO‐IP assay revealed association between endogenous RACO‐1 and YAP in EC9706 cells. EC9706 cells were harvested with RIPA lysis buffer. CO‐IP was performed using antibody as indicated. B, RACO‐1 increased YAP overall poly‐ubiquitination. HEK293 cells were transfected with 0.5 µg Myc‐RACO‐1 or Myc vector. After 24 h, cells were transfected with 1 µg HA‐Ub plasmid. After another 24 h, the cell extracts were immunoprecipitated with HA antibody. The poly‐ubiquitinated YAP was detected via Western blotting analysis. C, RACO‐1 increases K48‐linked poly‐ubiquitination of YAP. HEK293 cells were transfected with 0.5 µg Myc‐RACO‐1 or Myc vector. After 24 h, cells were transfected with 1 µg HA‐K48‐Ubi plasmid. After another 24 h, the cell extracts were immunoprecipitated with HA antibody. The K48‐linked poly‐ubiquitinated YAP was detected via Western blotting analysis. D, RACO‐1 decreases K63‐linked poly‐ubiquitination of YAP. HEK293 cells were transfected with 0.5 µg Myc‐RACO‐1 or Myc vector, together with 1 µg HA‐K48 Ubi plasmid. The cell extracts were immunoprecipitated with HA antibody. The K48‐specific poly‐ubiquitinated YAP was detected via Western blotting analysis

We performed more experiments to uncover the underlying mechanism between YAP and RACO‐1. Co‐immunoprecipitation showed the endogenous association between RACO‐1 and YAP in ESCC cells (Figure 5A). Nuclear and cytoplasmic separation based on CO‐IP showed that RACO‐1 interacts with YAP in the nuclear (Figure S1A‐B). As RACO‐1 is an E3 ubiquitin ligase, RACO‐1 could possibly modulate YAP stability via the ubiquitin‐dependent manner. The ubiquitin‐based immunoprecipitation assay in HEK293 cells showed that RACO‐1 overexpression could significantly increase YAP overall poly‐ubiquitination (Figure 5B). In order to detect whether YAP is degraded inside the nucleus. We used leptomycin B (LMB), a specific inhibitor of nuclear export, treated cells for 6 hours on the basis of the CHX assay, YAP was stabilized in the nucleus exposed to LMB, and the result showed that LMB treatment significantly decreased YAP half‐life (Figure S2A,B), and the ubiquitinated YAP was also improved when the transfected cells were treated with LMB (Figure S2C). Thus, we further extracted the cytosol and nuclear for the ubiquitin‐based immunoprecipitation assay, the experiment showed that RACO‐1 ubiquitinated YAP in the nuclear (Figure S1C). YAP‐5SA is a mutation of the five phosphorylation sites of YAP plasmid. We tested YAP ubiquitination level in YAP‐5SA condition, excluding the phosphorylation effect, and the result showed that RACO‐1 inducing YAP ubiquitination is Hippo‐independent (Figure S2D). There are some studies illustrated that K48‐linked ubiquitination promoted the degradation of YAP, whereas K63‐linked ubiquitination enhanced YAP nuclear localization and trans‐activation function.^19^ We further analysed the K48‐linked ubiquitination and K63‐linked ubiquitination in HEK293 cells. The data showed that RACO‐1 could promote K48‐linked ubiquitination of YAP, whereas RACO‐1 inhibited K63‐linked ubiquitination of YAP (Figure 5C‐D).

## 4 DISCUSSION

**Figure 6:**
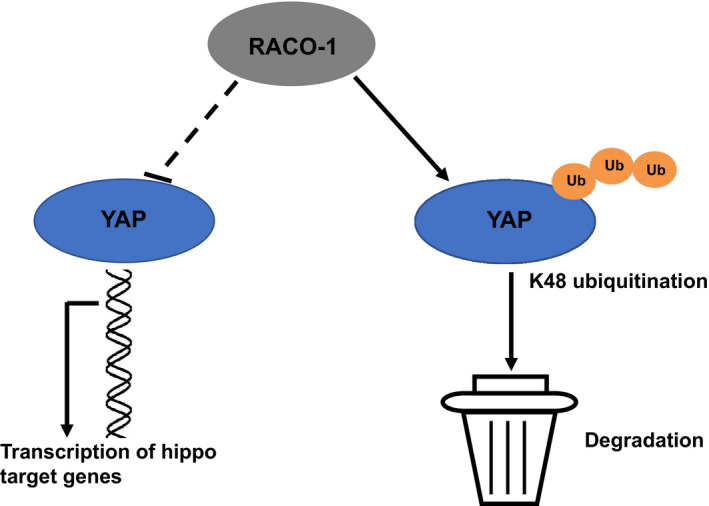
The hypothetical model for RACO‐1 regulating Hippo signalling in oesophageal squamous cell carcinoma: RACO‐1 protein associated with YAP and promoted YAP degradation via inducing YAP K48‐linked poly‐ubiquitination and inhibiting YAP K63‐linked poly‐ubiquitination

Our study showed that RACO1 as an ubiquitin ligase promotes YAP K48‐linked ubiquitination and proteasome‐dependent degradation in ESCC cells (Figure 6). Besides, RACO‐1 inhibits ESCC cell migration and invasion via the suppression of Hippo/YAP signalling. Our study provides a novel mechanism between RACO‐1 and Hippo signalling, which may be a hopeful marker for cancer diagnostics and therapeutics.

Previous studies identified that YAP/TEAD axis of Hippo signalling contributed to carcinogenesis of server human cancers.^11^ For example, YAP gene amplification was found in liver cancer, triple‐negative breast cancer and ESCC.^13^, ^20^ Besides, YAP/TAZ could trans‐activate many transcriptional factors, including RUNX, TEAD and AP1 family members.^10^, ^21^, ^22^ YAP mediated quite a few cancer phenotypes, such as migration, invasion, ‘stemness’ and EMT (epithelial‐mesenchymal transition) in human cancers. In oesophageal cancer, the dysregulation of Hippo signalling, such as YAP gene amplification and FAT mutations were found in 40% of all oesophageal cancer samples.^4^ YAP was identified that it could be associated with late cancer stage and poor survival in all oesophageal cancer patients.^13^ YAP depletion significantly decreased ESCC cell migration and invasion ability and tumour growth in vivo.^6^ Based on the significant function of Hippo/YAP in ESCC, targeting Hippo signalling may be an effective method to cure oesophageal cancer.

The TEAD interaction domain, WW domain and transcriptional activation domain constitute the three functional domains of YAP. The WW domain mediated the subcellular localization, whereas the TEAD‐binding domain associated with several transcriptional factors, including TEAD.^23^ YAP is the key mediator of Hippo signalling transduction and subject to several post‐translational modifications, such as ubiquitination and phosphorylation.

This pathway is mainly carried out by the cascade reaction of kinases, in which the upstream regulatory elements MST1 and MST1/2 phosphorylate and activate LATS1/2, the latter further phosphorylates the core effector factor YAP/TAZ, which leads to the degradation of YAP in the cytoplasm. For example, LATS1/2 can promote the phosphorylation of YAP at multiple sites (S127, S381), thus promoting the binding of YAP to 14‐3‐3 proteins.^24^ This action causes YAP to remain in the cytoplasm and leads to protein degradation.

In contrast, recent studies have shown that ubiquitin modification of YAP also plays an important role in Hippo signal regulation. For example, the SCF^β‐TRCP^ complex can interact with the YAP to facilitate its proteasome‐mediated degradation.^25^ In addition, FBW7, as a RING E3 ubiquitin ligase, can also cause ubiquitination and degradation of YAP at the k48 site.^26^ Our current study indicated that the RING E3 ligase RACO‐1 promotes ubiquitination and degradation of YAP at the k48 site. This novel finding provides a novel insight into the understanding of E3 ubiquitin ligase in control Hippo/YAP signalling activation by ubiquitin‐dependent manner.

RACO‐1 protein is composed of RING domain and IRES domain.^27^ RING domain is responsible for catalytic function of E3 ligation, whereas IRES domain functions to interaction with the substrates. One of the important finding is that RACO‐1 involves the regulation of RAS‐AP1 signalling.^14^ The activation of MEK‐ERK pathway promotes the K63‐linked ubiquitination of RACO‐1, which inhibits K48‐linked ubiquitination of CDC2 and Cylin D1 and their degradation.^14^ Besides, RACO‐1 is also the co‐factor of AP1 family members activation and cell proliferation. This could give the explanation that in our study why siRACO‐1 could inhibit cell proliferation in ESCC cells. Although several studies showed that RACO‐1 could potentially be an oncogene, our understanding is that RACO‐1 function is cancer type dependent.^15^, ^17^, ^18^ Our data showed that RACO‐1 could inhibit ESCC cell invasion via inhibition Hippo signalling. This could be the first study showing the tumour suppressive function of RACO‐1 in cancer progression. The interesting findings increase the proteolytic regulation of Hippo/YAP signalling, but also reveal the ‘multi‐face’ role of RACO‐1 in different cancer type backgrounds.

In conclusion, our study identified an endogenous inhibitor RACO‐1 of Hippo/YAP signalling in ESCC. RACO‐1 depletion could facilitate cancer cell invasion via activation of Hippo/YAP pathway in various of ESCC cell lines. As a newly discovered Hippo signalling regulator, regulating the activity or expression of RACO‐1 may be a feasible strategy for the treatment of ESCC patients.

## CONFLICT OF INTEREST

The authors have no conflict of interest.

## AUTHORS' CONTRIBUTIONS

LXM: Design and evaluation of concept of the study; supervision of the study; critical revision of the manuscript. WLD: Design and evaluation of concept of the study; supervision of the study; critical revision of the manuscript. CTM: Design and evaluation of concept of the study; evaluation; supervision of the study; critical revision of the manuscript. PD: Conduction of experiments; draft of the manuscript. WWL: Conduction of experiments; draft of the manuscript. ZXF: Conduction of experiments; draft of the manuscript. LK: Conduction of experiments. CZ: Conduction of experiments. WSJ: Conduction of experiments. HJH: Conduction of experiments. LBJ: Conduction of experiments. GC: Conduction of experiments. YZY: Conduction of experiments. HYH: Conduction of experiments. ZJH: Data analysis; data interpretation. CZG: Data analysis; data interpretation. WLC: Data analysis; data interpretation. SFF: Data analysis; data interpretation. LXM: Supervision of the study; critical revision of the manuscript. All authors: Manuscript reading; approval of the manuscript.

## ETHICAL APPROVAL

No ethic issues were involved in the study.

## Supporting information

Fig S1‐S2

## Data Availability

Additional data and materials may be requested from the corresponding author on reasonable request.
