# Supplementary figures and images for: RACO‐1 modulates Hippo signalling in oesophageal squamous cell carcinoma
Source: J Cell Mol Med. 2020 Sep 7;24(20):11912–21. doi: 10.1111/jcmm.15811 (PMC7579699; doi:10.1111/jcmm.15811)

Supplementary figure 1

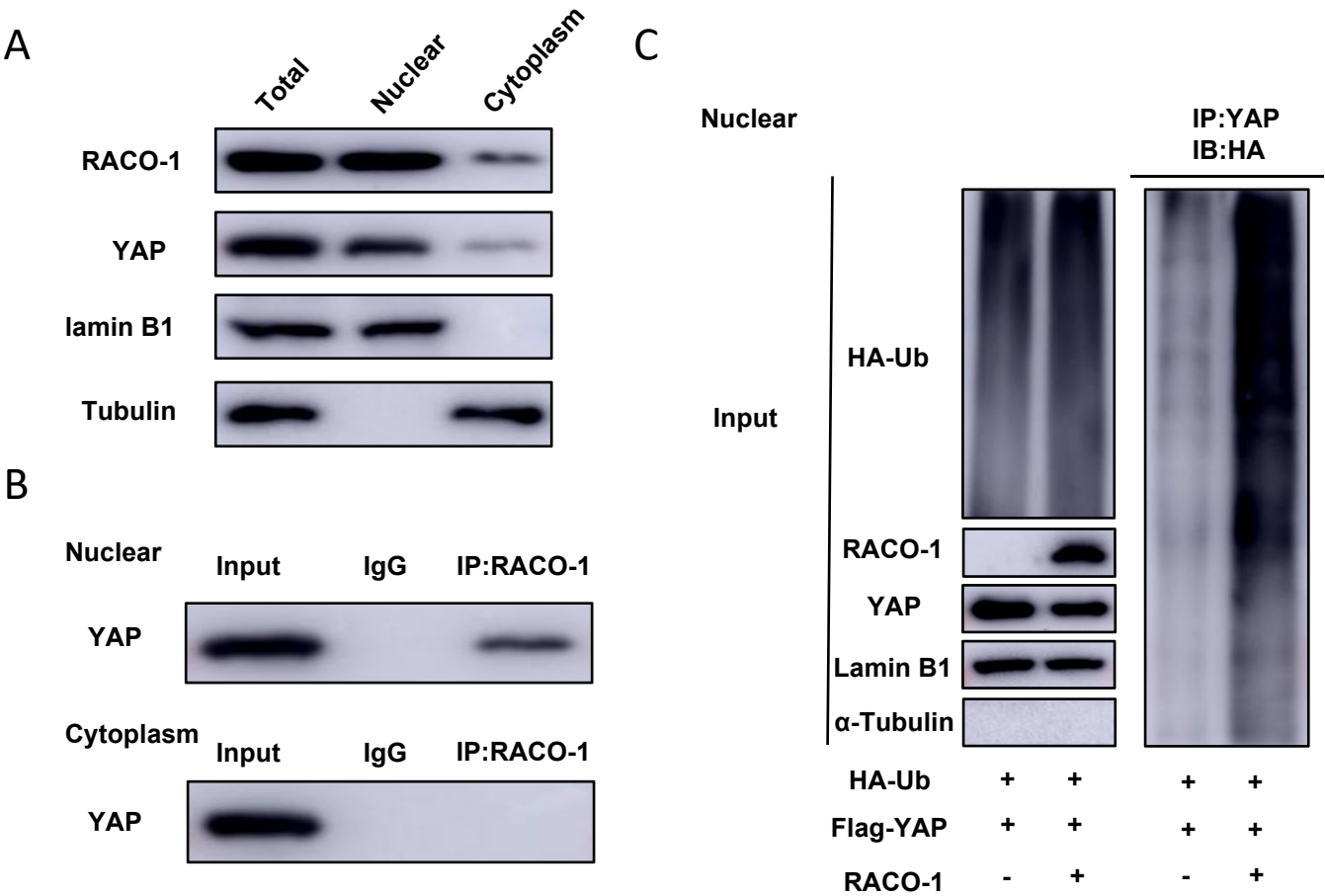

Supplementary figure 2

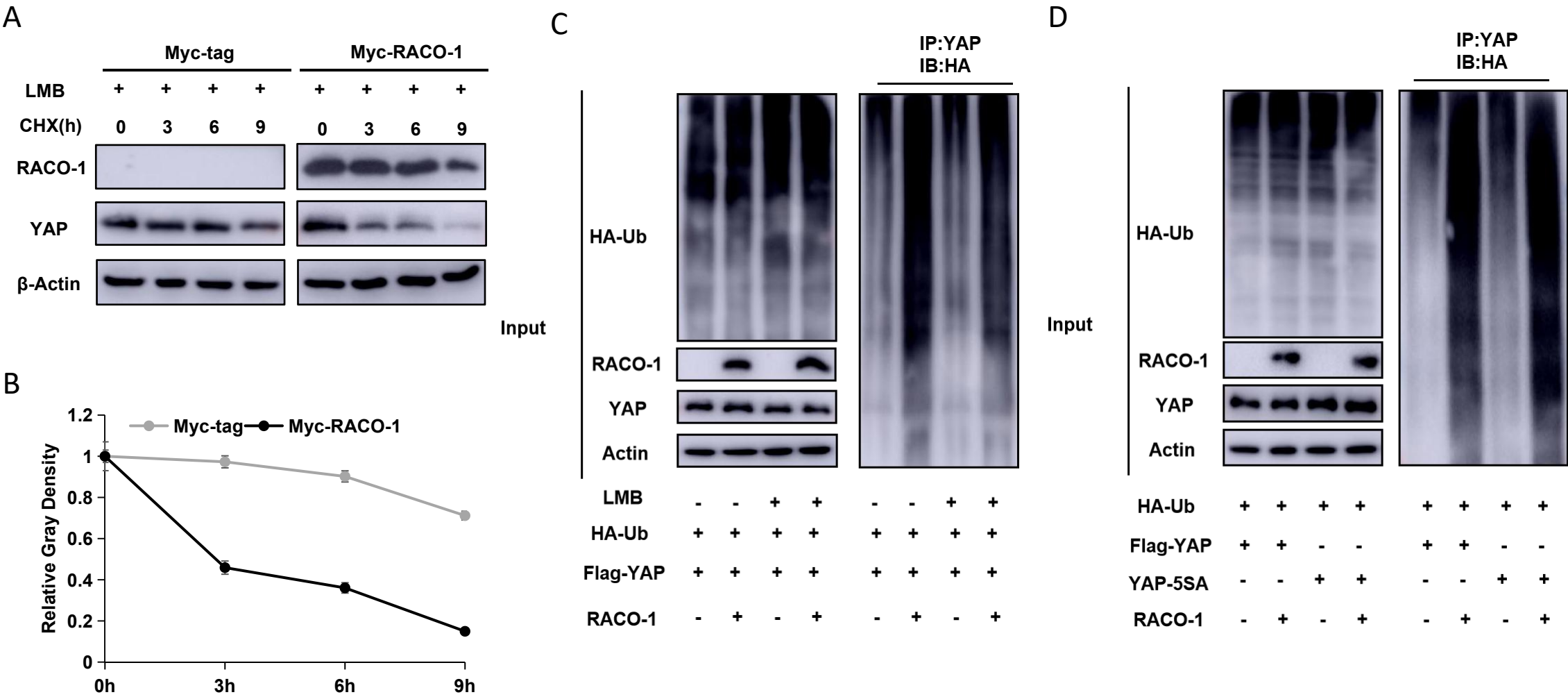

Supplement: Supplementary file 1 — Fig S1‐S2 [file JCMM-24-11912-s001.pdf]
